# How the Gastric Juice of the Horse’s Stomach Is Secreted

**Published:** 1883-10

**Authors:** 


HOW THE GASTRIC JUICE OF THE HORSE'S STOMACH IS SECRETED.—Drs. Ellenberger and Von Hofmeister have been making some very elaborate investigations into the physiology of a stomach digestion in the horse. Some of their conclusions we have already published. In the *Archiv für wissenschaftliche a practische Thierheilkunde* the final conclusions from their studies are given and we present them below.

First—The horse's stomach, which is comparatively small (holding about fifteen quarts), is divided into the Proventriculus which has no glands and the glandular stomach proper. The glandular stomach is filled with little pouches or follicles, and these are of two kinds, the mucous follicles or glands which secrete mucous and the peptic follicles or glands which secrete pepsin.

The entire stomach wall is very rich in elastic tissue, and this tissue surrounds even the glands themselves. There are nerve-ganglia in the stomach wall in its muscular and submucous coats.

Second—The so-called peptic glands are lined at their entrance with the cuboidal epithelial cells continued from the coating of the stomach. Within the glands or follicles themselves are two kinds of cells, the "chief-cell" (Hauptzell) and the "border-cell" (Belagzell). The latter is larger and seems to fill up the follicle like apples in a long bag.

Third—There is, besides in the horse, a third "wandering-cell" which seems to be in character and form between the two just mentioned. This cell has not been described as occurring in the dog's stomach.

Fourth—The glands in the pyloric part of the stomach which are generally thought to be mucous in function are lined with one kind of cell only which does not (as in man) resemble the "chief cells" in the peptic glands, but seem to be simply continuations of the epithelium lining the inner surface of the stomach. They are a little larger and granular.

Fifth—The surface epithelium and that lining the ducts of the glands secretes mucous.

Sixth—There are no lymph-follicles present in the stomach wall. But adenoid tissue exists, and the lymphatics are numerous.

Seventh—The portion of the stomach in which the pepsin is secreted is small, but very thick, and it possesses long follicles with unusually narrow ducts.

Eighth—The pyloric part of the stomach secretes no pepsin, or very little. (In this differing from the stomach of the dog.)

Ninth—The peptic glandular membrane secretes a great deal of pepsin, chiefly in its deeper parts.

There seems to be plenty of pepsin in the horse's stomach all the time, whether the organ be at rest or work. In this respect it also differs from the stomach of the dog and probably of man.

